# P-1729. Current trends on antifungal prophylaxis in solid organ transplantation

**DOI:** 10.1093/ofid/ofaf695.1900

**Published:** 2026-01-11

**Authors:** Jon Salmanton-Garcia, alessandro giacinta, maddalena gianella, Antonio vena, Patricia Muñoz, Oliver A Cornely, Maricela Valerio

**Affiliations:** University Hospital Cologne, Cologne, Germany, Cologne, Nordrhein-Westfalen, Germany; Department of Clinical Microbiology and Infectious Diseases, Hospital General Universitario Gregorio Marañón, Madrid, Spain, madrid, Madrid, Spain; Infectious Diseases Unit, IRCCS-Sant'Orsola Polyclinic, Bologna, Italy, bologna, Emilia-Romagna, Italy; Infectious Diseases Unit, San Martino Policlinico Hospital, IRCCS for Oncology and Neurosciences, Genoa, Italy; Department of Health Sciences (DISSAL), University of Genoa, Genoa, Italy, Genova, Liguria, Italy; Hospital General universitario Gregorio Marañón, Madrid, Spain; University of Cologne, Faculty of Medicine and University Hospital Cologne, Cologne, Nordrhein-Westfalen, Germany; Hospital General Universitario Gregorio Marañón. Clinical Microbiology and Infectious Diseases Department. Instituto de Investigación Sanitaria Gregorio Marañón, Madrid, Madrid, Spain

## Abstract

**Background:**

Invasive fungal infections (IFI) pose significant risks to solid organ transplant recipients, especially within the first 180 days post-transplantation. Current European and US guidelines are limited and lack strong evidence. Prophylactic strategies are moving away from universal approaches due to risks like rare fungal infections, adverse effects, drug interactions (especially with immunosuppressants), and higher costs. This study aims to outline current antifungal (AF) prophylaxis practices in solid organ transplant institutions and establish guidelines for managing IFI in this vulnerable patient population.

**Methods:**

From May 2023 to May 2024, tertiary care institutions were invited to participate in an online questionnaire on AF prophylaxis following solid organ transplantation. The survey gathered data on transplant volumes, incidence of IFI by pathogen, and prophylactic strategies, including triggers, preferred AF, and duration.

**Results:**

We analyzed 64 responses from 32 countries, primarily in Europe. Kidney transplants were most frequent, followed by liver transplants, often involving multi-organ procedures. Air quality measures, including air sampling and HEPA filter usage, varied widely, with lung and heart transplant units showing higher adoption rates. AF prophylaxis was consistently used in lung transplants and frequently in liver, bowel, and heart transplants, triggered by factors such as reintervention, organ retransplantation, or *Candida* spp. colonization. Preferred AF agents varied by organ type, commonly including liposomal amphotericin B, caspofungin, and fluconazole. Breakthrough IFI incidence varied significantly by organ type and specific pathogens, with the majority of institutions reporting dedicated infectious disease teams serving their transplant units.

**Conclusion:**

This survey provides a comprehensive overview of current AF prophylaxis practices in solid organ transplantation across diverse institutions. Addressing variability through standardized, evidence-based guidelines is essential for optimizing patient outcomes in managing IFI risks post-transplant.

**Disclosures:**

Jon Salmanton-Garcia, MSc, MPH, PhD, menarini, gilead, astrazeneca, pfizer: Honoraria Oliver A. Cornely, Prof. Dr., Al-Jazeera Pharmaceuticals/Hikma: Honoraria|Basilea: Advisor/Consultant|Cidara: Advisor/Consultant|Cidara: Board Member|Cidara: Grant/Research Support|Elion: Advisor/Consultant|F2G: Grant/Research Support|Gilead: Advisor/Consultant|Gilead: Grant/Research Support|Gilead: Honoraria|GlaxoSmithKline: Advisor/Consultant|GlaxoSmithKline: Honoraria|Grupo Biotoscana/United Medical/Knight: Honoraria|Melinta: Advisor/Consultant|Melinta: Board Member|MSD: Honoraria|Mundipharma: Advisor/Consultant|Mundipharma: Grant/Research Support|Mundipharma: Honoraria|Pfizer: Advisor/Consultant|Pfizer: Grant/Research Support|Pfizer: Honoraria|Pulmocide: Board Member|Scynexis: Advisor/Consultant|Scynexis: Grant/Research Support|Shionogi: Advisor/Consultant|Shionogi: Honoraria

